# Modified BLUE protocol ultrasonography can diagnose thrombotic complications of COVID‐19 with normal lung ultrasound

**DOI:** 10.1002/ccr3.4075

**Published:** 2021-03-28

**Authors:** Tamer Mohamed Zaalouk, Zouheir Ibrahim Bitar, Ossama Sajeh Maadarani, Ragab Desouky Ragab Elshabasy

**Affiliations:** ^1^ Critical Care Unit Ahamdi Hospital Kuwait Oil Company Ahmadi Kuwait; ^2^ Critical Care Unit Ahmadi Hospital Kuwait Oil Company Ahmadi Kuwait

**Keywords:** COVID‐19, focus echocardiography, modified BLUE protocol, venous thromboembolism

## Abstract

The BLUE protocol provides an excellent step‐by‐step approach for diagnosis of acute dyspnea. Adding FECHO (Focused Echocardiography) to the BLUE protocol completes the picture and helps make solid diagnoses, especially in submassive and massive PE (Pulmonary embolism). COVID‐19 infection can present with thrombotic manifestations like DVT (Deep vein thrombosis) and PE with no ultrasonographic evidence of lung parenchymal affection.

## Graphical abstract

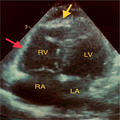

Acute dyspnea is one of the most distressing complaints for patients and one of the most challenging diagnoses for physicians. In many cases, physical examination and chest X‐ray alone are not helpful in diagnosis. Lung ultrasonography is becoming a standard tool in critical care medicine. The BLUE protocol of lung ultrasound is very beneficial, is easy to apply, and helps to save time that can be used for sophisticated investigations like computerized tomography examination of the chest. Anterior lung sliding is checked first. Its presence excludes pneumothorax. The B profile (anterior bilateral B‐lines associated with lung sliding) suggests pulmonary edema.

The A profile (anterior bilateral A‐lines associated with lung sliding) prompts a search for venous thrombosis. The presence of lung A profile plus venous thrombosis (Figure 1) puts pulmonary embolism as the cause of acute dyspnea at the top of the diagnosis list.

**FIGURE 1:**
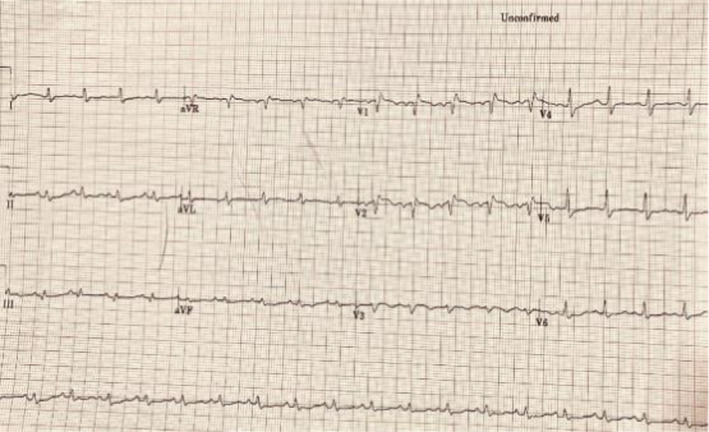
Electrocardiography (ECG) showing sinus tachycardia and RBBB

Extending the BLUE protocol of lung ultrasound by adding focused cardiac ultrasound (FECHO) can confirm the diagnosis of pulmonary embolism in this situation, especially in the presence of acute right ventricular strain pattern (Figure 2), pulmonary hypertension, and positive McConnell's sign (Figure 3).

**FIGURE 2:**
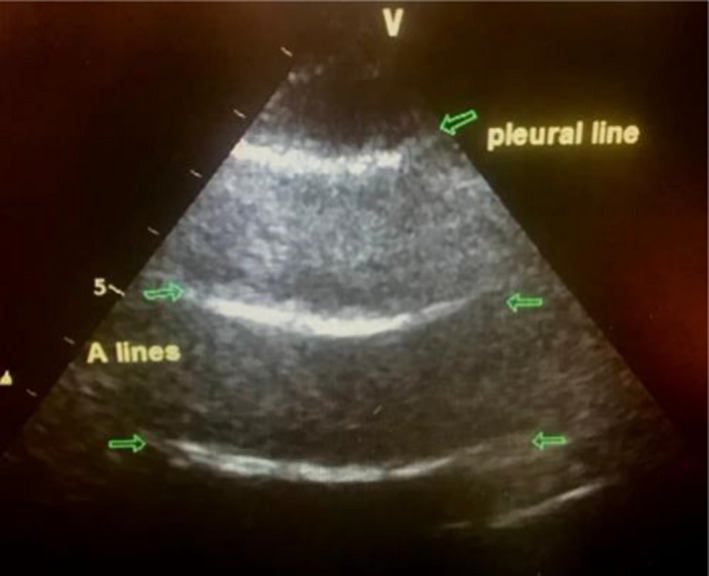
A profile lung

**FIGURE 3:**
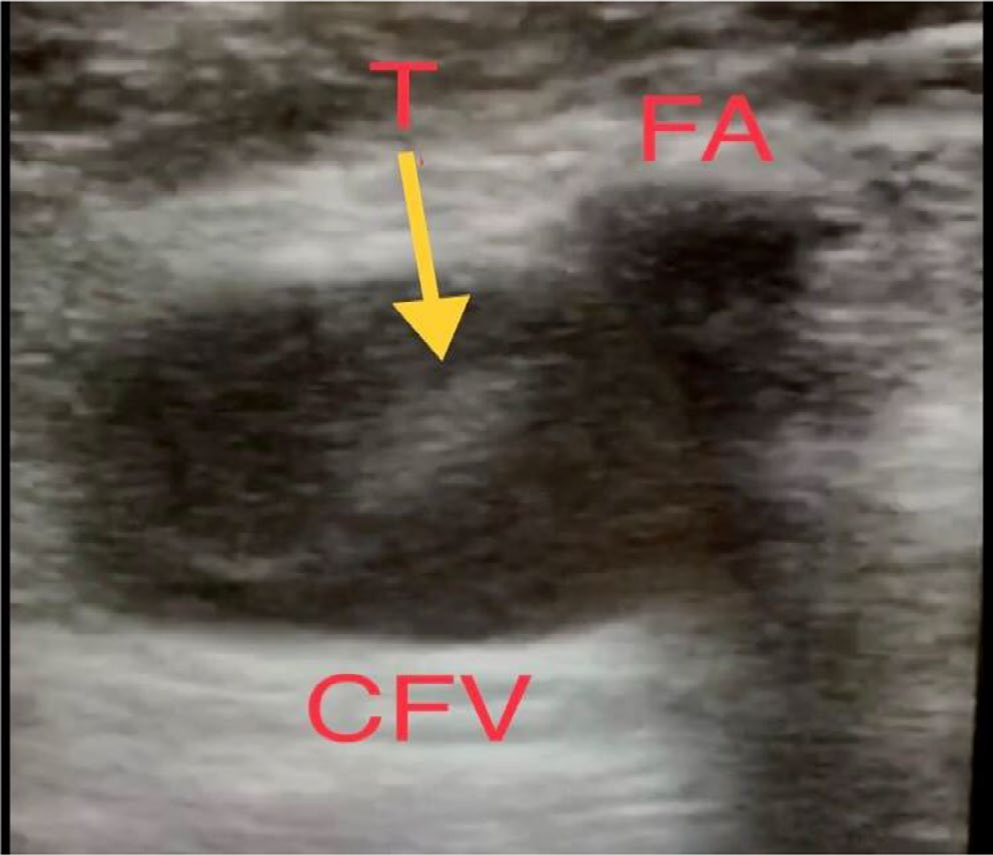
Doppler venous system of noncompressible right common femoral vein partial thrombosis (T). CFV, common femoral vein; FA, femoral artery; T, thrombus

In the current coronavirus disease 2019 (COVID‐19) pandemic, there are a lot of atypical presentations, especially thrombotic manifestations. We present a case of acute dyspnea with history of fever diagnosed by BLUE protocol ultrasonography plus FECO as a case of lower limb thrombosis and acute pulmonary embolism with no ultrasonographic evidence of lung parenchymal affection.

With the help of the modified BLUE protocol, we diagnose DVT and massive pulmonary embolism as manifestations of COVID‐19, which was confirmed later with a positive nasopharyngeal swab.

## 1 CASE REPORT

**FIGURE 4:**
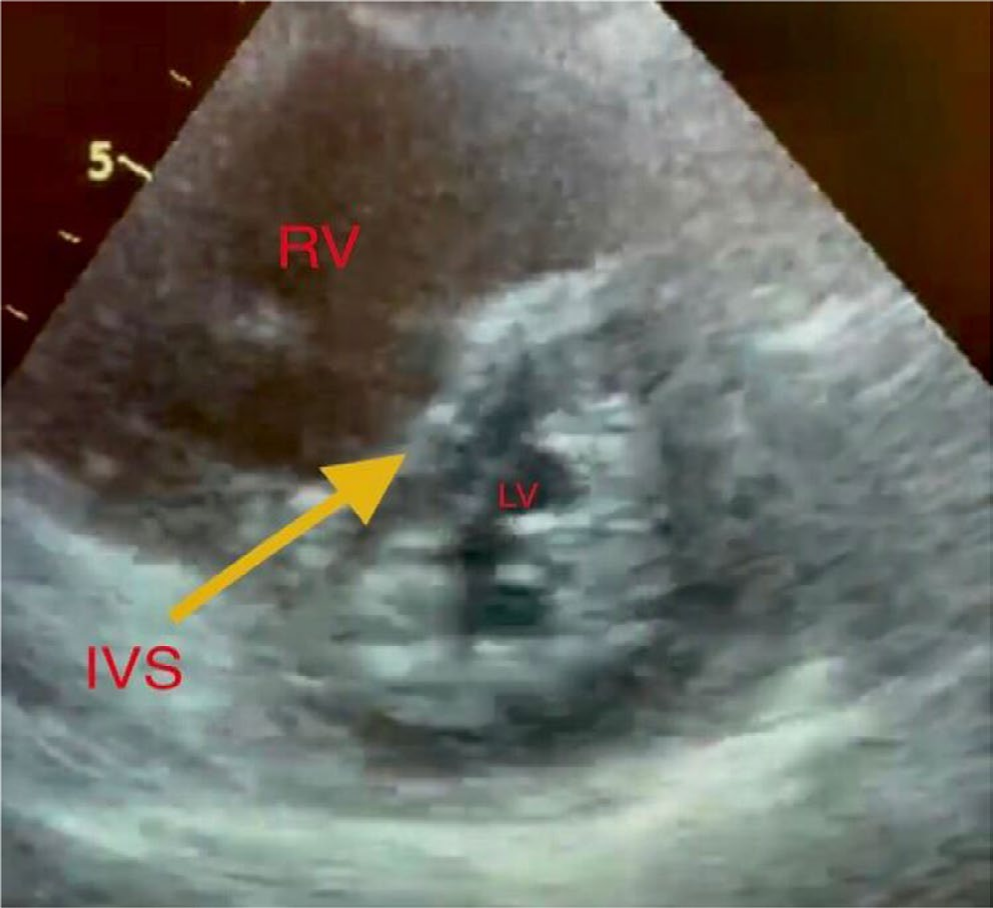
Parasternal short‐axis view showing dilated right ventricle (RV) with interventricular septum (IVS) shifted toward small left ventricle (LV)

A 71‐year‐old‐woman with past history of chronic kidney disease presented to the ER with acute dyspnea for 3 hours' duration. The patient had a history of low‐grade fever 3 days before presentation, no cough, no expectoration, and no hemoptysis. The patient was fully conscious but tachypneic (respiratory rate 24 breaths/min); pulse oximetry showed 90% on room air. Blood pressure was 90/50 mm Hg, and heart rate was 110 b/min. Chest X‐ray was normal, while electrocardiography (ECG) showed sinus tachycardia with a new right bundle branch block (Figure 4).

**FIGURE 5:**
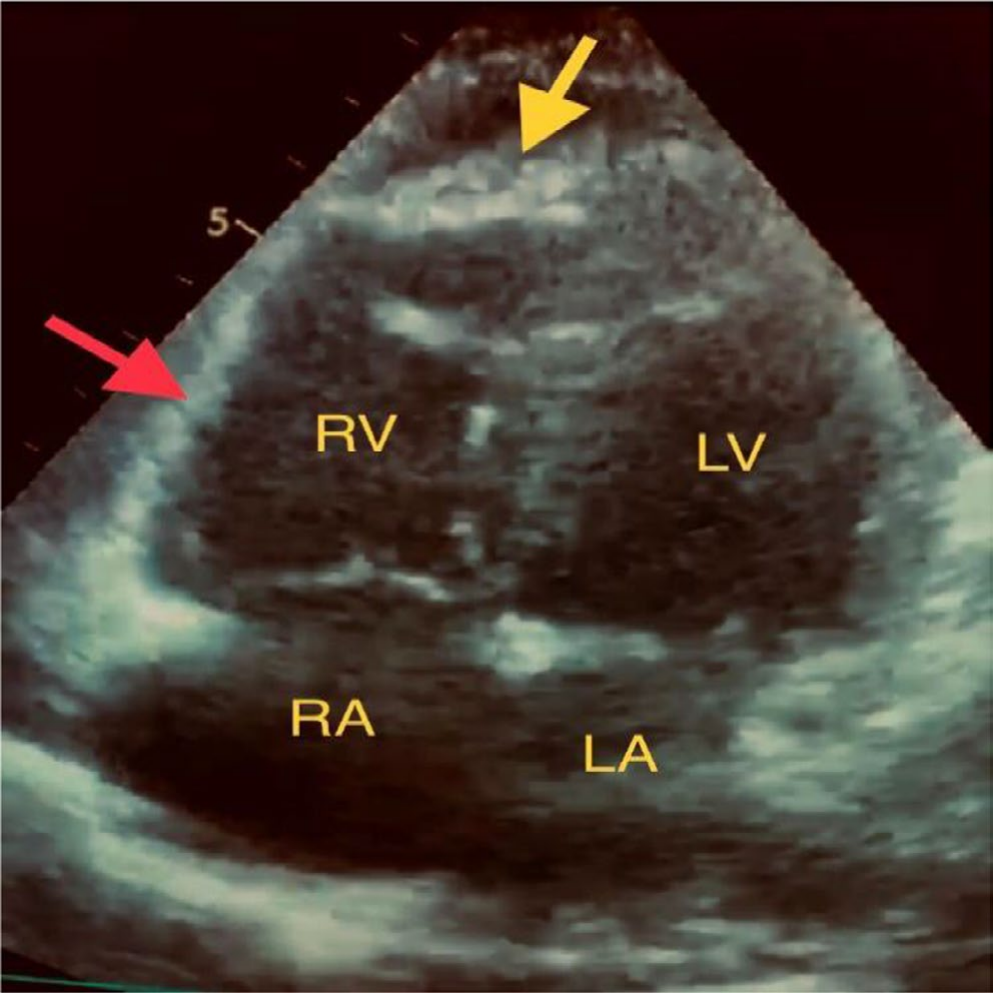
McConnell's sign: Akinesia of mid‐free wall of right ventricle (red arrow) with normal motion at the apex (yellow arrow). RV, right ventricle; LV, left ventricle; RA, right atrium; LA, left atrium

Arterial blood gas analysis in ambient air confirmed type 1 respiratory failure (PaO_2_ 9.4 kPa). As a case of acute dyspnea, the BLUE ultrasound protocol was applied bedside, finding that there was normal lung sliding with an A profile of the lung ultrasound (Figure 5), no B‐lines, and no subpleural consolidations.

According to the BLUE protocol sequences, we proceeded to assess lower limb veins, and unfortunately, there was bilateral femoral vein thrombosis. At this point, the main differential diagnosis was pulmonary embolism (PE). We modified the BLUE protocol by adding focused cardiac ultrasound (FECHO) assessment, which confirmed the diagnosis of PE, as it showed right ventricular dilation, positive McConnell's sign, and pulmonary hypertension (pulmonary artery systolic pressure equal to 50 mm Hg). Such LUS and FECHO findings during the current pandemic raised the suspicion of COVID‐19 infection as a cause of the patient's clinical presentation.

Laboratory findings were significant for mild leukocytosis with lymphopenia, raised D‐dimer, troponin I, and pro‐BNP, and there was evidence of acute kidney injury.

## 2 LABORATORY RESULTS

**Table:** 

Blood tests results
WBCs (white blood cell count) = 15 000
HB (hemoglobin) = 13.4 g
Platelets = 103,000
Urea = 15 mg/dL
Creatinine = 2 mg/dL
ALT = 61.9 U/L AST = 55.1 U/L GGT = 109 U/L CRP = 47.3 mg/L D‐dimer = 5 Troponin I = 100 Pro‐BNP = 10 000

The patient was started on intravenous fluids and high‐flow O_2_, and a nasopharyngeal swab for SARS‐CoV‐2 was taken. She remained hypotensive and hypoxic. Thrombolytic therapy according to the PE protocol was administered, and the patient was intubated and mechanically ventilated due to progressive hypoxia and hypotension. The nasopharyngeal swab for SARS‐CoV‐2 returned positive. Unfortunately, the patient remained hypoxic and died after a few hours.

## 3 DISCUSSION

Acute dyspnea could be a common symptom within the ED. The standard approach to dyspnea often relies on radiologic and laboratory results, causing excessive delay before adequate therapy is started. Use of an integrated point‐of‐care ultrasonography (POCUS) approach can shorten the time needed to formulate a diagnosis while maintaining a suitable safety profile.^1^

Chest computerized tomography (CT) has significant limitations, like exposure to ionized radiation; limited application in certain patients, like pregnant women; the need to transfer a potentially unstable patient to the tomography unit; and being time‐consuming.^2^

Point‐of‐care ultrasonography is employed by physicians at the bedside for rapid, focused, and accurate evaluation to spot or rule out various pathologies. Several protocols for POCUS are currently available and employed in different clinical scenarios like undifferentiated dyspnea, hypoxia, or shock and include the bedside lung ultrasound in emergency (BLUE), rapid assessment of dyspnea with ultrasound (RADIUS), and rapid ultrasound in shock (RUSH) protocols.^3^

The bedside lung ultrasonography in emergency (BLUE) protocol is an algorithm developed by Lichtenstein as a systemic approach to the diagnosis of patients with dyspnea in critical care units (ICUs) with 90.5% diagnostic accuracy. The BLUE protocol provides a good step‐by‐step approach to diagnose acute dyspnea.^4^

The BLUE protocol starts with checking for anterior lung sliding. Presence of sliding excludes pneumothorax. The B profile suggests pulmonary edema. The A profile prompts an enquiry into thrombosis. The association of the A profile with phlebothrombosis (venous scan) favors the diagnosis of pulmonary embolism with 81% sensitivity and 99% specificity.^5^

At this point, adding a focus ECHO (FECHO) examination to the BLUE protocol can confirm the presence of embolism (PE), especially if it is massive or submassive PE. Ultrasound signs of right heart strain include bowing of the IVS into the LV, right ventricular dilatation and systolic dysfunction including McConnell's sign, possible tricuspid regurgitation, a dilated inferior vena cava, and visual right heart thrombus.^6^

Coronavirus disease 2019 (COVID‐19) is caused by severe acute respiratory syndrome coronavirus 2 (SARS‐CoV‐2). The World Health Organization (WHO) declared it a pandemic on 11 March 2020.^7^

The effect of SARS‐CoV‐2 on endothelial cells plays a vital role in vascular injury that contributes to pulmonary, cardiovascular, and other manifestations endothelial dysfunction and endothelitis are considered the idea of thrombus formation and lead to COVID‐19–associated thromboembolic insult to various organs and might partially explain the hypercoagulable state commonly related to patients infected with SARS‐CoV‐2.^8^, ^9^

Thromboembolic manifestations of COVID‐19 have been described in several reports. In the ED and critical care units, the BLUE protocol plus FECHO in the appropriate clinical context is an effective tool to rapidly diagnose acute pulmonary embolism associated with right heart strain and possible thrombus in transit and to guide further treatment.^10^

In our case, the patient presented with acute dyspnea with no clinical symptoms or signs that favored diagnosis of COVID‐19. However, using an extended BLUE protocol by adding FECHO helped in diagnosing acute massive PE with bilateral lower limb DVT. Within the context of the present pandemic with thrombosis everywhere, we diagnosed this patient with COVID‐19 infection, which was confirmed by positive nasopharyngeal swab for SARS‐CoV‐2 shortly thereafter.

We stress the importance of the BLUE protocol for assessment of patients with acute dyspnea and recommend including FECHO examination for more accurate diagnosis.

## 4 CONCLUSION

COVID‐19 infection can present with thrombotic manifestations like DVT and PE with no ultrasonographic evidence of lung parenchymal affection. The BLUE protocol provides an excellent step‐by‐step approach for diagnosis of acute dyspnea. Adding FECHO to the BLUE protocol completes the picture and helps make solid diagnoses, especially in submassive and massive PE.

## CONFLICT OF INTEREST

The author(s) declare no potential conflicts of interest with respect to the research, authorship, and/or publication of this article.

## AUTHOR CONTRIBUTIONS

TZ: wrote the article, ZIB and OSM shared in the discussion and, with RE, in collecting the data and revision of the manuscript. Our working website is www.kockw.com (Kuwait Oil company, Ahamdi Hospital).

## CONSENT

Informed consent was obtained from the patient for the publication of this case report.

## Data Availability

The data that support the findings of this study are available from the corresponding author upon reasonable request.
